# Perceived body image in men and women with type 2 diabetes mellitus: correlation of body mass index with the figure rating scale

**DOI:** 10.1186/1475-2891-8-57

**Published:** 2009-12-16

**Authors:** Harold E Bays, Debbra D Bazata, Kathleen M Fox, Susan Grandy, James R Gavin, SHIELD Study Group

**Affiliations:** 1 Louisville Metabolic and Atherosclerosis Research Center, Louisville, KY, USA; 2 St. Luke's Primary Care South, Overland Park, KS, USA; 3 Strategic Healthcare Solutions, LLC, Monkton, MD, USA; 4 AstraZeneca LP, Wilmington, DE, USA; 5 Emory University School of Medicine, Atlanta, GA, USA

## Abstract

**Background:**

Body mass index (BMI) is often used as an objective surrogate estimate of body fat. Increased BMI is directly associated with an increase in metabolic disease, such as type 2 diabetes mellitus (T2DM). The Stunkard Figure Rating Scale (FRS) is a subjective measure of body fat, and self-perceptions of body image conceivably impact the development and treatment of T2DM. This study examined the self-perception of body image to various levels of BMI among those with T2DM.

**Methods:**

Respondents (n = 13,887) to the US ***S***tudy to ***H***elp ***I***mprove ***E***arly evaluation and management of risk factors ***L***eading to ***D***iabetes (SHIELD) 2006 survey self-reported their weight and height for BMI calculation. On the gender-specific Stunkard FRS, respondents selected the figure most closely resembling their body image. Spearman correlation was computed between perceived body image and BMI for men and women separately. Student's *t*-test analysis compared the mean BMI differences between respondents with and without T2DM.

**Results:**

Men with T2DM did not significantly differ from men without diabetes mellitus in mean BMI per body image figure except at the extremes in body figures. Women with T2DM had a significantly higher BMI for the same body figure compared with women without diabetes mellitus for most figures (p < 0.05).

**Conclusions:**

Individuals, particularly women, with T2DM may differ in their perception of body image compared with those without diabetes mellitus. It is unclear if these perceived differences increase the risk of T2DM, or if the diagnosis of T2DM alters body image perceptions.

## Introduction

Body fat is often estimated by an objective surrogate measure of body mass index (BMI), which is determined by height and weight, and reported in units of kilogram/meter squared (kg/m^2^). Body fat can be subjectively assessed by the Stunkard Figure Rating Scale (FRS) [1], which utilizes gender-specific body figures. Both types of measurements are useful to assess the relationship of body weight to adverse clinical outcomes [2,3].

Increasing BMI is associated with an increased prevalence of metabolic diseases [4], and BMI is a common objective parameter reported in clinical trials of patients with type 2 diabetes mellitus (T2DM), as well as in clinical trials of patients with hypertension, dyslipidemia, and obesity. The FRS has often been used to assess perceived body images among those of differing ages [2,5], genders [2-7], racial and ethnic groups [2,8-10], religion [11], and countries [12,13], as well as among those with psychological/psychiatric conditions, such as eating disorders [2,3,14]. Perception of body image may influence health-related behaviors such as physical activity, nutrition, and other behaviors which are important components of T2DM management. If an overweight individual perceives him/her self as disproportionately thinner compared with actual BMI, then he/she may be less understanding of the need for weight reduction. Other studies have examined perception of self-reported weight [15] and desired body image [16] among individuals with T2DM; but they did not examine the correlation between body image and BMI among adults with and without T2DM. No prior study has evaluated the relationship of a gender specific FRS in T2DM patients and their correlation with self-reported BMI. The objective of the present study was to examine the correlation between the Figure Rating Scale figures and BMI among individuals with and without diabetes mellitus.

## Methods

A cohort of individuals with a diagnosis of T2DM and a cohort without diabetes mellitus were selected from the **S**tudy to **H**elp **I**mprove **E**arly evaluation and management of risk factors **L**eading to **D**iabetes (SHIELD), the largest survey study of its kind. SHIELD included an initial, cross-sectional, self-reported, screening questionnaire to identify areas of interest in the general population (which included individuals with T2DM). The baseline survey evaluated prior diagnoses, health status, health knowledge, attitudes, behaviors, and treatment. Annual follow-up self-reported surveys to the same recipients evaluated changes in behaviors, treatment, and health status. A detailed description of the SHIELD methodology was previously published [17,18].

In brief, in 2004, a screening survey was mailed to a stratified random sample of 200,000 US households, representative of the US population for geographic residence, household size and income, and age of head of household [19]. The head of household provided responses to the screening survey for up to 4 adult (aged ≥ 18 years) household members, resulting in a response rate of 63.7% (127,420 households for 211,097 adults). The baseline survey was sent to a representative sample of individuals (n = 22,001) who were identified in the screening survey as having type 1 diabetes mellitus or type 2 diabetes mellitus or being at risk for diabetes mellitus. Each respondent group was balanced to be representative of that population for age, gender, geographic region, household size, and income for the U.S. population, and then a random sample from each group was selected and sent the baseline survey. A response rate of 72% was obtained. Since 2005, yearly SHIELD surveys captured self-reported information on health status, attitudes and behaviors, quality of life, and anthropometry from this sample. This analysis is based upon a cross-sectional sample, evaluating the relation between BMI and the Stunkard FRS, which was first included in the 2006 SHIELD survey (n = 13,877). The response rate for the 2006 survey was 75%.

Respondents were categorized as having T2DM based upon self-report of having "ever been told by a doctor, nurse, or other healthcare professional that you have diabetes; if yes, which type (type 1, type 2, or gestational)". T2DM was defined as a physician diagnosis of T2DM and age of onset >21 years of age since some respondents did not know the type of diabetes mellitus, and because setting a threshold age of onset was thought to increase the accuracy of a diagnosis of T2DM. While conceivable that some respondents greater than 21 years of age may not have known that they had T1DM, and while T1DM may rarely occur after age 21, the authors concluded that the chances of misdiagnoses using these criteria were small. No diabetes mellitus was defined as no physician diagnosis of type 1 diabetes mellitus, T2DM, or gestational diabetes mellitus. Respondents with gestational diabetes mellitus or type 1 diabetes mellitus were excluded from the analysis.

### Study measures

BMI (weight/height^2 ^expressed in kg/m^2^) was based upon self-report of height and weight. The validated [20] Stunkard FRS [1] was based on subjective self-selection of body image figures. The FRS consists of two gender-specific scales that contain nine schematic figures of women and nine figures of men, ranging from underweight to overweight. On this gender-specific scale, respondents selected a figure that most closely resembled their body image. For men, the scale of body figures ranged from 1 to 9, with 1 being the thinnest body type and 9 being the largest, most obese type. For women, the scale of body figures ranged from 10 to 18, using the same range of thinnest to largest as the men's scale.

Each participant was provided a measuring tape with their survey and given written instructions to while standing, hold the tape measure loosely around their waist at the level of their belly button to measure waist circumference. Use of the umbilicus was thought to be more easily understood and provide consistent data across respondents.

### Statistical analyses

Spearman correlation was computed between perceived body image and calculated BMI for men and women separately. Student's *t*-test analysis compared the mean BMI differences between respondents with T2DM and without diabetes mellitus. Ordered logistic regression model was constructed to control for differences in age, gender, race, income and education. The ordered logistic regression was used since the figures have a natural ordering (low to high) but the distances between the adjacent levels are unknown. The Figure Rating Scale figures were scored as 1-9 for men and 1-9 for women. Statistical significance was set *a priori *as p < 0.05.

## Results

For men responding to the FRS, 1,304 respondents had T2DM and 2,924 had no diabetes mellitus. For women responding to the FRS, 1,979 respondents had T2DM and 4,763 had no diabetes mellitus. T2DM respondents had greater mean BMI, greater mean age, generally less education, and lower household incomes and were less likely to be white than respondents with no diabetes mellitus (Table 1).

**Table 1:** Characteristics of SHIELD respondents with type 2 diabetes mellitus or no diabetes mellitus

Characteristics	T2DM (n = 3,283)	No diabetes mellitus (n = 7,687)
**Age, years, mean (SD)**	61.3 (12.3)*	55.5 (16.2)
**Males, %**	39.7	38.0
**White, %**	85.5*	89.0
**Education, % with no more than a high school degree**	35.2*	28.6
**Income, % with <$35,000**	45.9*	34.9
**BMI for men, mean (SD)**	32.1 (7.1)*	30.1 (6.0)
**BMI for women, mean (SD)**	35.5 (9.0)*	30.4 (7.7)
**Waist circumference for men, cm, mean (SD)**	112.9 (20.0)*	107.4 (16.7)
**Waist circumference for women, cm, mean (SD)**	112.8 (19.8)*	99.7 (19.4)

*p < 0.001 vs. no diabetes mellitus

**Figure 1:**
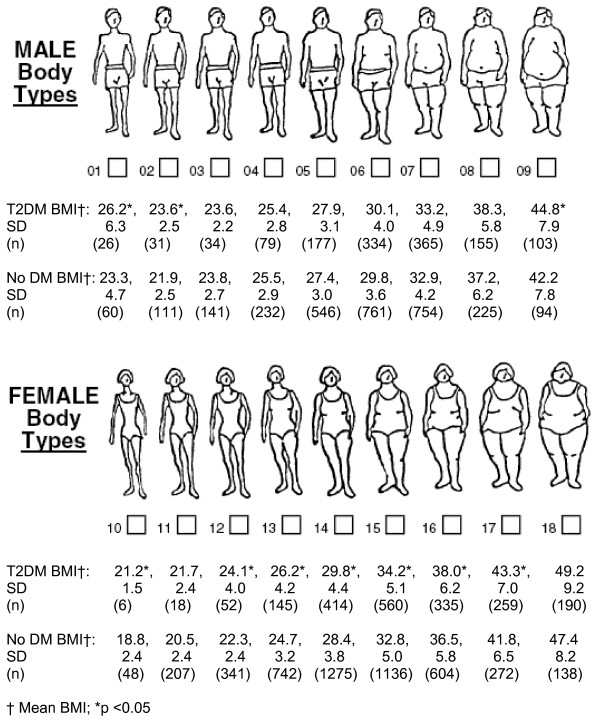
**Body image figures and mean body mass index (BMI) for men and women with and without diabetes**. DM = diabetes mellitus; T2DM = type 2 diabetes mellitus.

**Figure 2:**
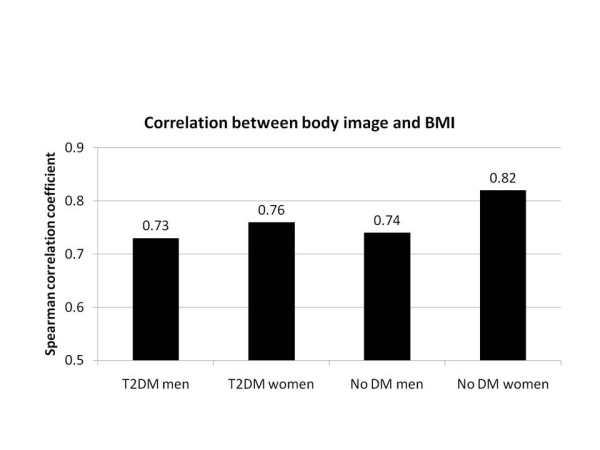
**Correlation between Figure Rating Scale and body mass index (BMI)**. Correlation significant for each group (p < 0.0001). DM = diabetes mellitus; T2DM = type 2 diabetes mellitus.

Based upon their mean BMI, men with T2DM did not significantly differ in their selection of FRS body figure compared with men without diabetes mellitus, except at the extremes in body image figures (Figure 1). For body figures 1, 2, and 9, men with T2DM had significantly higher mean BMI compared with men without diabetes mellitus (p < 0.05). Women with T2DM had a significantly higher BMI for the same body image figure compared with women without diabetes mellitus (p < 0.05) at all body figures except for figures 11 and 18. For figures 11 and 18, women with T2DM had higher mean BMI compared with women without diabetes mellitus but the difference was not statistically significant (p > 0.05).

Self-perception of body image as assessed with the FRS for T2DM and no diabetes mellitus groups was significantly correlated with BMI for men and women (p < 0.0001) (Figure 2). Correlation coefficients ranged from 0.73 to 0.74 for men and 0.76 to 0.82 for women, showing strong correlation.

The ordered logistic regression controlling for differences between the groups, confirmed the correlation analysis by demonstrating a significant association between BMI and the FRS figures (p < 0.0001). The proportional odds for a one unit increase in BMI on the Figure Rating Scale was 1.38 (95% CI = 1.37 - 1.39) given that age, gender, race, income and education were held constant in the ordered logistic regression model.

## Discussion

In the largest self-reported survey study of its kind, the SHIELD data revealed that compared with men without diabetes mellitus, men with T2DM generally did not significantly differ in their selection of a body image on the FRS, based upon similar mean BMI, except at the extremes in body image. In contrast, women with T2DM generally had a higher BMI for each body figure that they felt best reflected their appearance compared with women without diabetes mellitus.

Limitations of this study include potential selection bias since the SHIELD survey was a mailed survey but the response rate was very high for a mailed survey (75%). Also, household panel surveys, like SHIELD, tend to under-represent the very wealthy and very poor segments of the population and do not include military or institutionalized individuals [21,22]. Another concern is that patients may not accurately self-report measurements such as height and weight. However, other studies have indicated that such self-reported measurements are accurate [23,24]. Also, the high correlation between BMI and body image might not mean that the respondents' perception is close to their actual BMI. Respondents could consistently underestimate or overestimate BMI and still have a high correlation with body image. Regarding the self-reporting of metabolic disease, prior analyses have demonstrated generally good correlation between the prevalence of T2DM as assessed by SHIELD when compared with the prevalence of T2DM determined by objectively measured surveys such as US NHANES [4,25]. This is likely, in large part, because the diagnosis of diabetes mellitus is dependent upon a single parameter (glucose) that is generally known and frequently measured.

Also, the FRS may have limitations due to scale coarseness and constant height across the different figures [26]. However, this scale is one of the most widely used assessment tools in body image and psychometric research [5-7,9,10] and reported to be valid and reliable [20]. Approximately, 20% of SHIELD respondents did not answer the body image question; however, the respondents with missing data were not significantly different from those who did respond. Finally, while perceived body images may vary among those of differing ages, racial and ethnic groups, countries, and psychological profiles, no adjustments were made for these parameters in this analysis since the study population was largely Caucasian, all respondents were from the U.S., and of similar age range.

Self-reported survey data have advantages in specific circumstances. A main component of this analysis included the FRS. As opposed to the generally objective BMI, the FRS is entirely subjective. As such, ascribing BMI to individual figures in the FRS cannot be done solely by objective analysis. Rather, the only manner to derive subjective data is to ask individuals to provide their perceptions. A self-reported survey completed within a home environment may be a more "objective" way to determine subjective data, in that it is possible that individuals may be more comfortable, and thus more honest, in selecting body images than might occur in a clinical setting.

The importance of the findings of this study is at least 2-fold. Firstly, given the large number of respondents, this may represent the best available data in assigning BMI to individual FRS figures for T2DM. A review of the literature reveals limited information as to what BMI correlates to individual FRS figures in men and women, with no prior similar analysis of this size having been published for T2DM. One prior study attempted to establish BMI norms for the FRS [27] and included twins and their family members, mostly from Virginia, USA. The present study found higher BMI levels for each body figure for men and women without diabetes mellitus than observed in the Bulik twin study [27]. This difference may be partially because the present study respondents included non-twin individuals from across the US. The findings of this current analysis were, however, similar to other studies that have examined self-perception of body weight. For example, a study of patients receiving care from general practitioners in Australia [15] found that a large proportion of overweight and obese patients did not perceive themselves as being overweight based on self-reported weight.

Secondly, this is the first study to suggest that there are discrepancies in body image among individuals with T2DM, at least in women. The reasons for these discrepancies are unclear. Even though this study found a strong correlation between BMI and body image perception, misperception of one's own weight-related appearance is common [13]. Previous studies suggest that body image may be a risk factor for obesity [14,28]. One could speculate that it is a discrepancy of body image perception that might contribute to excessive body weight, and thus an increased risk for T2DM. Another possibility is that it is not a discrepancy of perceived body image that precedes the diagnosis of T2DM. Instead, it may be that after diagnosis of T2DM, patients may then develop an altered perception of body image. Once an individual is diagnosed with T2DM, little doubt exists that the patient's life changes in the form of altered insurance rate status, interaction with family and friends, increased doctor visits (including routine eye examinations, foot examinations, etc), more frequent laboratory testing, and greater evaluation and management of multiple risk factors, especially regarding nutrition, physical exercise, lifestyle, blood pressure, and lipids. It could be that these daunting life changes upon being diagnosed with T2DM might result in alterations in multiple health-related perceptions, including perceptions of body image compared with those without diabetes mellitus. It is possible that once diagnosed with T2DM and confronted with its associated health and cost burdens, patients may then place less emphasis on body image.

## Conclusion

Overweight individuals with T2DM may have different body image perceptions compared with overweight individuals without diabetes mellitus as observed with the larger body figures. This suggests potential opportunities for clinicians to incorporate the understanding of this unique challenge (and potential obstacle) in weight loss strategies. Some data suggest that no negative consequences (such as depression) are observed upon having and failing to meet weight loss expectations, even expectations that are thought to be unrealistic [29]. Nonetheless, if body image perception does have the potential to affect behavior, then having overweight T2DM individuals perceive their body image closer to their actual BMI may assist clinicians to better make the case for the need for weight reduction. Furthermore, because perception may influence behavior, the differences in body image perception among women with and without diabetes mellitus that were not observed among men suggest that overweight T2DM men and women may differ in optimal approaches and strategies directed at weight loss.

## Competing interests

SHIELD is supported by funding from AstraZeneca Pharmaceuticals LP. HEB and JRG and DDB are advisory board members for AstraZeneca Pharmaceuticals LP. KMF received research funds from AstraZeneca Pharmaceuticals LP to conduct the study. SG is an employee and stock holder of AstraZeneca Pharmaceuticals LP.

## Authors' contributions

HEB: participated in the design of the study and helped to draft the manuscript; DDB: participated in the design of the study and helped in reviewing the manuscript critically; KMF: participated in the study design and coordination, carried out the data analysis and drafted the manuscript; SG: conceived of the study, participated in the study design, acquired funds, supervised the data collection and helped in critically reviewing the manuscript; and JRG: participated in the study design and helped in critically reviewing the manuscript. All authors read and approved the final manuscript.
